# OLDER AND MORE MINDFUL? ASSOCIATIONS OF MINDFULNESS CHARACTERISTICS AND WELL-BEING VARY WITH AGE

**DOI:** 10.1093/geroni/igz038.3116

**Published:** 2019-11-08

**Authors:** Leeann Mahlo, Tim D Windsor

**Affiliations:** Flinders University, Adelaide, South Australia, Australia

## Abstract

Research examining how mindfulness confers benefits for well-being is in its infancy. Furthermore, few studies have considered the positive effects of mindfulness on psychological functioning from a lifespan perspective. The present study aimed to examine a recently proposed model of mindfulness and whether the importance of the proposed mechanisms for well-being varied as a function of age. A community-based sample of 623 participants aged between 18 and 86 years (M = 48.78, SD = 16.74) was recruited via an internet-based research platform. Participants completed questionnaire measures of mindful characteristics (i.e., present-moment attention, nonjudgment, interoception, acceptance, nonattachment, and decentering), flexible goal adjustment, and well-being. Parallel mediation analyses using bootstrapping showed that both present-moment attention and nonjudgment provided significant pathways to (a) well-being through acceptance, nonattachment, and decentering; and (b) flexible goal adjustment through nonattachment and decentering. Furthermore, most aspects of mindfulness were positively associated with age. Conditional process analyses revealed that the direct relationships between (1) present-moment attention and well-being, (2) nonjudgment and well-being, and (3) decentering and flexible goal adjustment were significant for adults from around age 40 and became stronger with increasing age. The findings provide preliminary support for a recently proposed model of mindfulness and suggest that present-moment attention, nonjudgment, and decentering may become especially important for well-being across the second half of life. In particular, these aspects of mindfulness may represent psychological qualities that require a relatively modest investment of physiological and cognitive resources and can be targeted in interventions designed to enhance well-being in later adulthood.

